# Understanding the Non-Steady Electrochemical Mechanisms of the Stress Corrosion Cracking of X70 Pipeline Steel in a Marine Environment

**DOI:** 10.3390/ma18092073

**Published:** 2025-04-30

**Authors:** Bo Xu, Baozhuang Sun, Yang Dai, Fei Xie, Feng Huang, Zhiyong Liu, Xiaogang Li

**Affiliations:** 1 Key Laboratory of Oil and Gas Storage and Transportation Technology, Fushun 113001, China; 2 Key Laboratory for Corrosion and Protection (MOE), Institute of Advanced Materials and Technology, University of Science and Technology Beijing, Beijing 100083, China; 3 China Petroleum Engineering & Construction Corporation Beijing Branch, Beijing 100083, China; 4 The State Key Laboratory of Refractories and Metallurgy, Wuhan University of Science and Technology, Wuhan 430081, China

**Keywords:** X70 pipeline steel, SCC, cathodic protection, hydrogen embrittlement, non-steady electrochemistry

## Abstract

In this study, a non-stationary electrochemical model was verified to be equally applicable to X70 pipeline steel under polarization potential in a marine environment, and the mechanism of stress corrosion cracking (SCC) was revealed. A quick SCC evaluation model for X70 pipeline steel in a marine environment was established. The model only requires electrochemical tests and a small number of slow strain rate tests to obtain the stress corrosion susceptibility distribution of pipeline steel across the whole potential range. The model is applicable to the marine environment and is characterized by its easy operation and accurate results.

## 1. Introduction

As offshore oil and gas exploration continues to advance rapidly, X70 pipeline steel has emerged as a critical material due to its excellent strength and cost-effectiveness, playing a pivotal role in marine resource development [1,2,3]. Nevertheless, its susceptibility to stress corrosion cracking (SCC) in harsh marine environments poses significant safety risks, threatening structural integrity and operational reliability [4,5,6]. Previous studies have provided valuable insights into the SCC behavior of various steels under marine conditions. For instance, Wu et al. [7] explored the effect of cathodic protection potentials on the SCC susceptibility of X80 pipeline steel, highlighting the interplay between anodic dissolution (AD) and hydrogen embrittlement (HE). Although their findings offered foundational knowledge on high-strength pipeline steels, direct applicability to X70 steel, with its unique microstructural and electrochemical properties, remains uncertain. Further studies addressing the SCC sensitivity of pipeline steels have emphasized the importance of cathodic protection (CP). Investigations by Sun et al. [8] on high-strength steel wires for cables under marine atmospheric conditions demonstrated that CP parameters critically influence corrosion rates. Additionally, Liu et al. [9] provided evidence of the complex hydrogen-related degradation mechanisms in X70 pipeline steel in simulated soil environments, underscoring the need for a detailed understanding of hydrogen evolution kinetics under varying CP conditions. However, these studies predominantly focus on specific environmental factors or particular steel types without sufficiently addressing the marine-specific electrochemical processes involved in SCC.

A variety of studies have aimed at elucidating the electrochemical mechanisms underlying the SCC susceptibility of steels in marine environments [10,11,12,13,14,15,16,17,18]. Ramamurthy and Atrens [10] comprehensively reviewed the SCC of high-strength steels, emphasizing the interplay of environmental conditions and material properties. Meanwhile, Sun et al. [11] specifically examined X70 steel exposed to marine sediments containing sulfate-reducing bacteria, highlighting microbially influenced corrosion under CP conditions. Nevertheless, the majority of these studies are descriptive in nature or isolated within narrow conditions, lacking a systematic approach that quantitatively evaluates SCC susceptibility across the entire range of cathodic potentials typically encountered in practical marine service. Previous quantitative assessments of SCC susceptibility, such as those developed for duplex stainless steel by Pan et al. [19] and E690 steel by Ma et al. [20], demonstrate the utility and necessity of establishing standardized, systematic evaluation methods. Sun et al. [21] further investigated X70 pipeline steel in simulated seawater, indicating a transition from oxygen reduction to hydrogen evolution reactions under varying dissolved oxygen contents. Chen et al. [22] utilized density functional theory (DFT) to show that X70 steel retains SCC susceptibility even under optimized CP conditions, highlighting the complexity of SCC phenomena. Despite these advances, a clear gap remains; no established quantitative methodology currently exists to rapidly assess the SCC susceptibility of X70 pipeline steel across the entire CP potential spectrum specific to marine environments.

To bridge this critical knowledge gap, the present study systematically investigates the SCC susceptibility of X70 pipeline steel under a comprehensive range of CP potentials in simulated seawater. By combining electrochemical impedance spectroscopy (EIS), slow strain rate testing (SSRT), and detailed fracture surface analysis, we aim to validate the applicability of non-steady-state electrochemical theory to marine environments and to establish a robust, practical model capable of accurately predicting SCC behavior in X70 steel. This study not only addresses the specific limitations highlighted by prior research but also provides essential guidance for ensuring the structural safety and reliability of pipeline steels in offshore engineering applications.

## 2. Materials and Methods

### 2.1. Materials and Solutions

**Figure 1:**
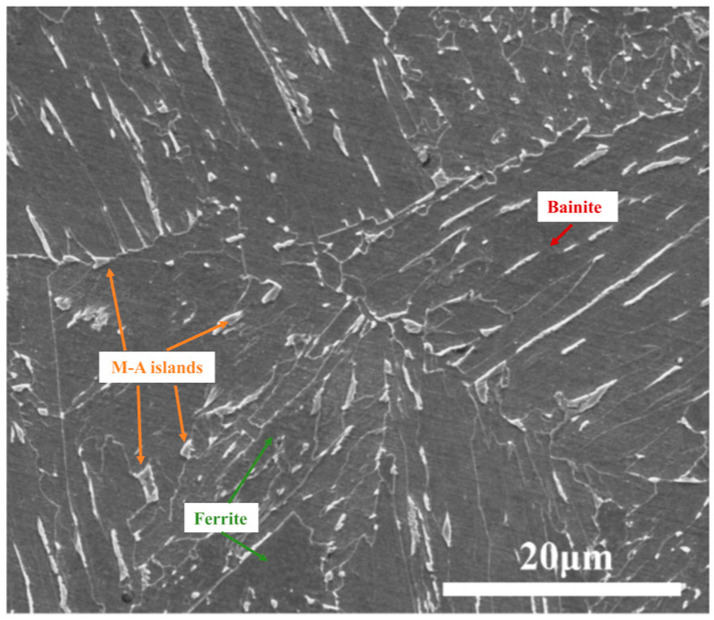
X70 metallographic organization.

X70 pipeline steel was used as the test material for this experiment. Its chemical composition is outlined in Table 1. Its mechanical properties are outlined in Table 2. The metallographic structure is illustrated in Figure 1. This shows a typical ferrite–bainite duplex structure, with the bainite showing a lamellar/needle-like morphology.

In this experiment, a simulated seawater solution was used as the test medium. The simulated seawater was based on relevant standards [23]. To simulate seawater conditions, nitrogen was injected into the solution to vary the dissolved oxygen level, which was maintained at 3.8 mg/L. Table 3 provides detailed information on the composition of the test solution.

### 2.2. Electrochemical Test

Copper conductors were soldered to the back of the X70 specimen for electrochemical testing. The specimens were sealed with epoxy resin and the exposed 10 mm x 10 mm surface was the electrochemical work surface. Prior to use, the work surface was sanded to 2000# with silicon carbide sandpaper in increments, then rinsed with deionized water and ultrasonically cleaned with anhydrous ethanol.

The experiments were carried out using a three-electrode system with the X70 steel specimen as the working electrode, a platinum sheet as the auxiliary electrode, and a silver/silver chloride electrode as the reference electrode, and the EIS measurements were carried out at cathodic potentials of OCP, −800 mV, −850 mV, −900 mV, −950 mV, −1050 mV, and −1200 mV (vs. Ag/AgCl/seawater). The polarization curves were scanned from −1.2 V to −0.2 V (vs. Ag/AgCl/seawater). The fast scanning speed was 50 mV/s and the slow scanning speed was 0.5 mV/s. The frequency range of the AC impedance test was 50 kHz to 10 mHz, the excitation potential was 10 mV, and all the experiments were carried out at 25 °C and repeated three times to ensure the consistency of the results.

### 2.3. SSRT Test

**Figure 2:**
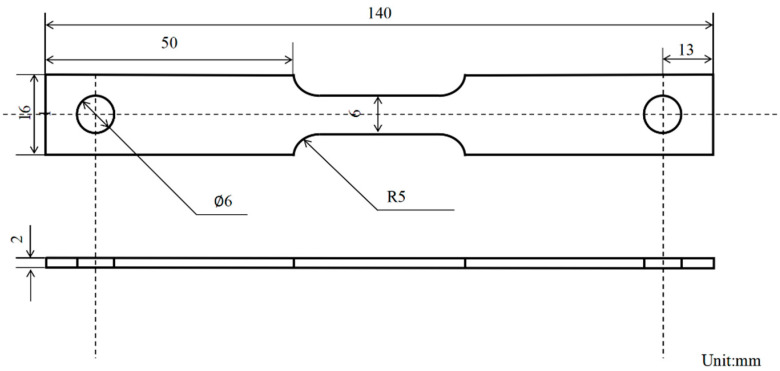
Tensile specimen schematic diagram.

**Figure 3:**
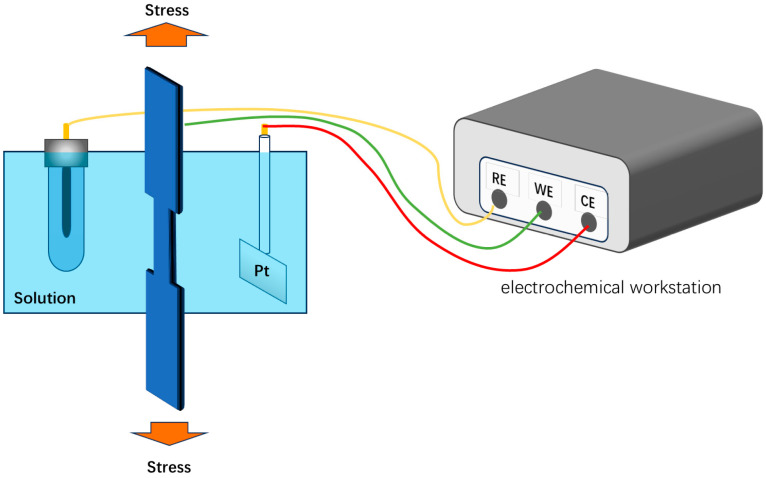
The schematic diagram of the stretching apparatus for this work.

SSRT tests were performed using a slow strain rate tensile machine (WDML-30 kN, LETRY, Xi’an, China) with a strain rate of 1 × 10^−6^ s^−1^. The specimen size was as shown in Figure 2, which was in accordance with the standards specified in relevant standards [24], and the tensile specimens were ground axially with silicon carbide sandpaper up to 2000#, and the specimens were immersed in the test solution for 12 h before the test, and the electrochemical workstation (CS353,Corrtest, Wuhan, China) was used to apply potentials to the specimens during the test process. During the test, a electrochemical workstation (CS353, Corrtest, Wuhan, China) was used to apply potentials to the specimens, and the experimental setup is shown in Figure 3. To ensure the reliability of the experimental results, all SSRT experiments were repeated three times under consistent conditions. Upon the completion of the experiments, measurements were taken for both the elongation and the specimen’s area reduction. $I_{\delta }\;$ and $I_{\psi }$ were calculated using established formulas to effectively quantify and characterize the stress corrosion susceptibility exhibited by the material [19].

(1) $$I_{\delta }=\left(1-\delta_{S}/\delta_{0}\right)\times 100\%$$

(2) $$\begin{array}{c} I_{\psi }=\left(1-\psi_{S}/\psi_{0}\right)\times 100\% \end{array}$$

where $I_{\delta }$ and $I_{\psi }$ denote the SCC susceptibility indices, which are quantified by the loss rate of elongation and the reduction rate of area, respectively. In this context, $\delta_{0}$ represents the elongation measured in air, whereas $\delta_{s}$ corresponds to the elongation observed at various potentials in the simulated seawater environment. Similarly, $\psi_{0}$ and $\psi_{s}$ indicate the reduction in the area measured in air and under different potential conditions in the simulated seawater environment, respectively.

After the SSRT test, the fracture surface was cut, rinsed with deionized water, and ultrasonically cleaned to remove corrosion products. The fracture morphology and secondary cracks were observed using a scanning electron microscope (FEI Quanta 450, Thermo Fisher Scientific, Waltham, MA, USA). The secondary electron (SE) mode was employed during the experiment, with the accelerating voltage set to 15 kV and the working distance (WD) set to 10 mm.

## 3. Results

### 3.1. Potentiodynamic Polarization Curve

**Figure 4:**
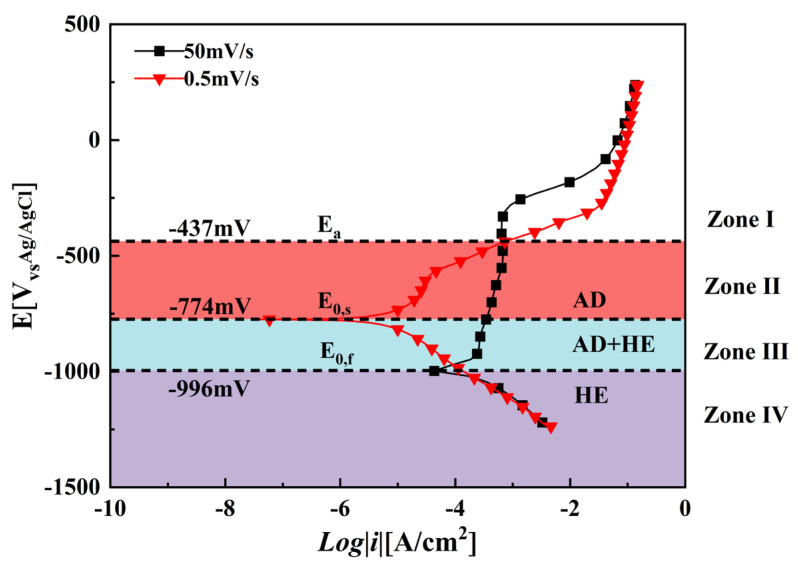
Polarization curves of X70 pipeline steel at different scan rates in simulated marine environments.

Figure 4 shows the potentiodynamic polarization curves for X70 pipeline steel, representing the results for both scanning conditions. In the case of the fast scan, the corrosion potential is about −996 mV and about 222 mV, which is more negative than the value recorded during the slow scan. The noticeable variation indicates a markedly greater electrochemical activity at the tip of the crack in contrast to the wall of the crack [25,26,27].

The variation in the polarization curves for the fast and slow scans enables us to categorize the differing potential ranges into four distinct regions, especially at −437, −774, and −996 mV, as shown in Figure 4. In Zone I, which region is beyond −437 mV, the AD current exhibits minimal variation between the two curves, exerting an almost negligible influence on crack growth, and consequently indicating a relatively low susceptibility to SCC. In Zone II, identified between −437 and −774 mV, the AD current at the crack tip is considerably greater than at the walls, greatly enhancing the likelihood of crack initiation and progression. Under these circumstances, the SCC is primarily facilitated by the AD mechanism. In Zone III, ranging from −774 to −996 mV, both the AD at the crack tip and the HE along the crack faces happen simultaneously. The interaction among AD, the presence of hydrogen, and tensile stress results in a mixed-mode SCC mechanism. Although both processes contribute to SCC in this zone, neither is particularly strong, which leads to only moderate susceptibility levels. Finally, in Zone IV, found below −996 mV, large hydrogen evolution takes place at the crack tip and along the crack wall, which markedly heightens SCC susceptibility and could lead to brittle fractures. This observation is consistent with numerous previous studies [28,29,30]. Liu et al. [12] showed that AD predominantly affects SCC when the potential exceeds the zero-current potential determined by the slow-scan technique; conversely, HE dominates at negative potentials above the zero-current potential derived from fast-scan measurements. In the interval between these two zero-current potentials, both the AD and HE mechanisms work together to regulate the SCC phenomenon.

### 3.2. EIS Tests

**Figure 5:**
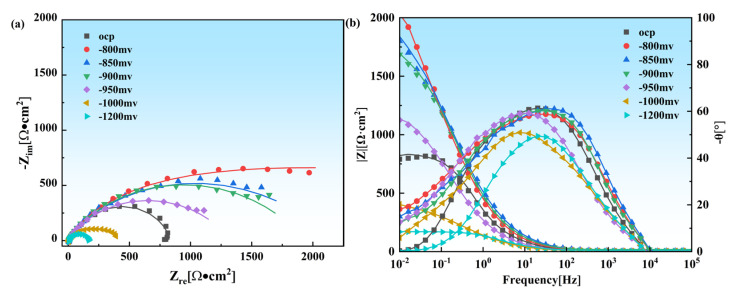
The electrochemical impedance spectra of X70 pipeline steel were investigated at OCP and various levels of cathodic polarization potentials within a simulated marine environment. (**a**) Nyquist plots; (**b**) Bode plots.

The EIS results for X70 pipeline steel in simulated marine environments at OCP and different applied potentials are shown in Figure 5. In the Nyquist plots, a shift to a more negative potential results in a preliminary enlargement of the capacitive arc radius, which peaks at around −800 mV before decreasing. This trend suggests that X70 pipeline steel experiences optimal protection at approximately −800 mV, effectively reducing AD. When the potential was further adjusted to −950 mV, the capacitive arc radius decreased but was still larger than the radius observed at OCP. This indicates that the cathodic processes gradually shift towards hydrogen evolution within this specific range of potentials, even though this evolution remains relatively moderate.

As the cathodic potential progresses toward increasingly negative values, particularly in the range of −1000 mV to −1200 mV, there is a notable and sharp decrease in the radius of the capacitive arc. This phenomenon strongly suggests that a vigorous HER is occurring at these specified potentials. At potentials where the cathodic reactions are primarily driven by oxygen reduction, the low-frequency impedance modulus, measured at 0.01 Hz, serves as an indicator of the corrosion resistance of X70 steel. However, when the HER becomes the dominant process, the low-frequency impedance modulus begins to correlate more directly with the rate of hydrogen evolution. When the potential transitions from the open-circuit potential (OCP) to −800 mV, the low-frequency impedance modulus exhibits an increase from approximately 8 × 10^2^ Ω·cm^2^ to around 1.9 × 10^3^ Ω·cm^2^. This increase is indicative of enhanced corrosion resistance in the material. Even at a cathodic potential of −950 mV, despite a decrease in the impedance modulus to about 1.08 × 10^3^ Ω·cm^2^, this value still remains above that observed at OCP. This observation points to the conclusion that corrosion is being effectively mitigated under these conditions, and the HER does not appear to be significant at this potential. In contrast, once the potential reaches −1200 mV, there is a rapid decline in the impedance modulus to roughly 1.7 × 10^2^ Ω·cm^2^, highlighting a substantial increase in the activity of the HER at this extreme potential.

**Figure 6:**
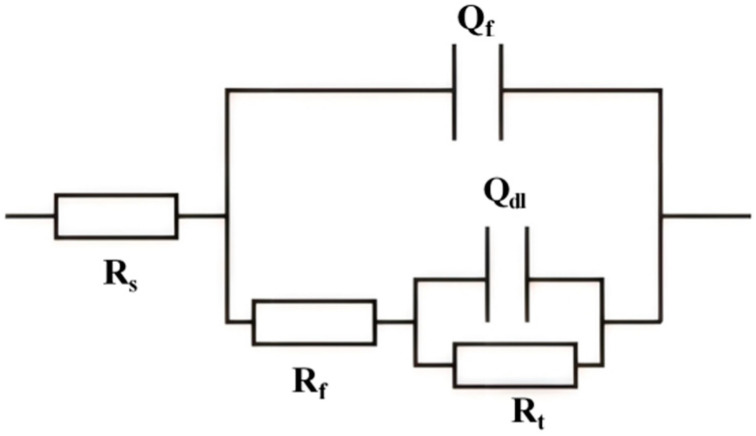
Equivalent circuit diagram of EIS.

**Figure 7:**
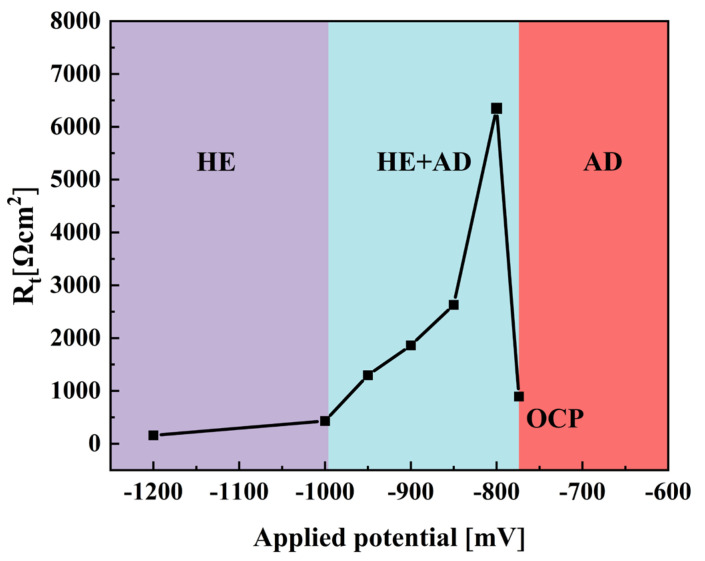
Dependence of R_t_ on potentials.

The Equivalent circuit diagram is shown in Figure 6. The electrochemical parameters, including solution resistance (R_s_), film resistance (R_f_), the film constant phase element (Q_f_), double-layer constant phase element (Q_dl_), and charge transfer resistance (R_t_), were determined by fitting the EIS data to an appropriate equivalent circuit model. Figure 7 shows the relationship between R_t_ and potential.

**Figure 8:**
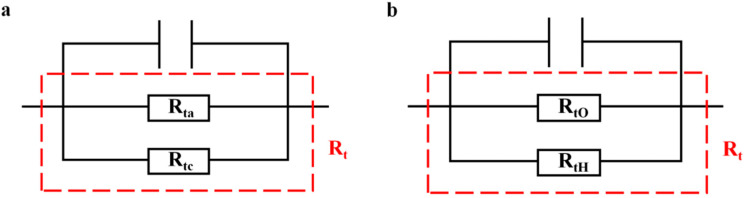
Equivalent circuit for EIS at (**a**) weak cathodic polarization and (**b**) strong cathodic polarization.

The R_t_ can be viewed as the parallel arrangement of the anodic charge transfer resistance (R_ta_) alongside the cathodic charge transfer resistance (R_tc_), as shown in Figure 8. When the potential becomes increasingly negative, the anodic overpotential diminishes, resulting in a significant rise in R_ta_, while the cathodic overpotential escalates, which causes a slight reduction in R_tc_. The cumulative impact of these alterations considerably elevates R_t_. At −800 mV, the cathodic reaction is mainly dominated by oxygen reduction, R_t_ achieves its peak value, with AD being minimal and the rate of hydrogen evolution remaining low. In these circumstances, the steel demonstrates improved corrosion resistance and effective HE prevention. As the potential is driven further negative towards −1200 mV, the HER becomes more pronounced, and the cathodic charge transfer resistance is effectively characterized as the parallel combination of oxygen reduction (R_tO_) and hydrogen evolution (R_tH_), as represented in Figure 8. The participation of hydrogen evolution has a significant impact on the kinetics of the cathodic reaction, leading to a decrease in overall R_t_ as the potential shifts from −800 mV to −1200 mV. These observations are consistent with the findings derived from the Bode plots, which confirm that at −800 mV, R_t_ is at its highest, AD is completely suppressed, and the system is chiefly controlled by oxygen reduction with negligible hydrogen participation.

### 3.3. SSRT Tests

**Figure 9:**
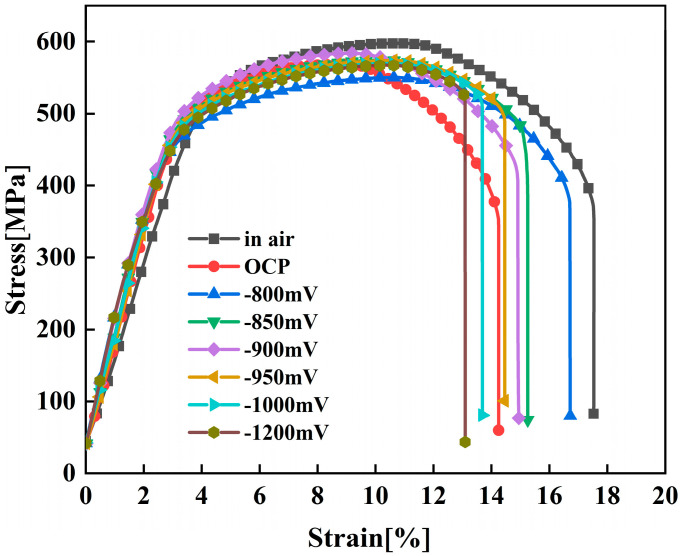
Stress–strain curve of X70 pipeline steel at various potentials in simulated seawater.

**Figure 10:**
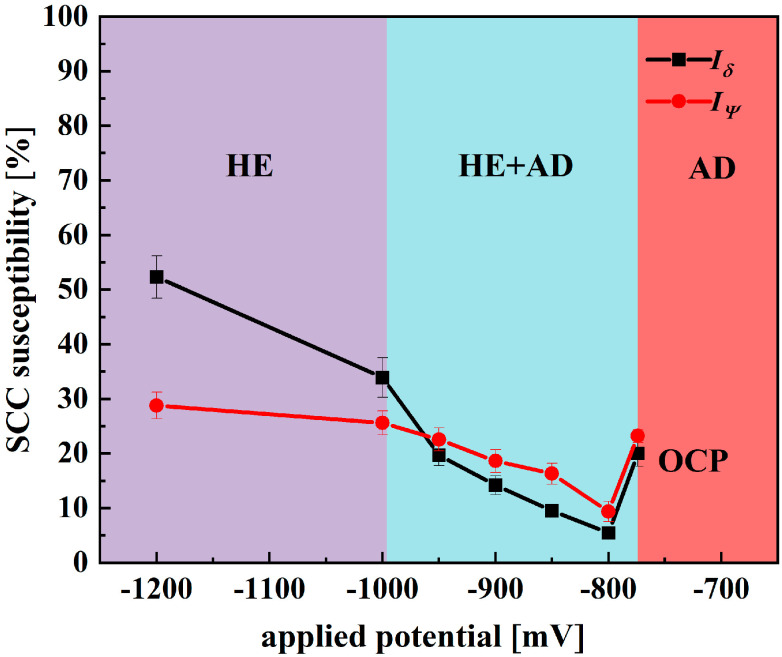
Dependence of SCC susceptibility on potentials.

Figure 9 shows the stress–strain curves of X70 pipeline steel when subjected to various applied potentials in a simulated seawater environment. From the figure, it can be seen that both the OCP and CP potentials, compared with those in air, present a certain stress corrosion susceptibility, and this stress corrosion susceptibility shows a tendency of decreasing first and then increasing with the increase in CP potential. Among them, at the −800 mV potential, the elongation and section loss rate are the highest, and the SCC susceptibility is the smallest. And, at the −1200 mV potential, the elongation and section loss rate are the lowest, and the SCC susceptibility is the highest. The difference in yield strength and ultimate tensile strength between OCP and various potentials was not significant. Mechanical strength was significantly lower at all potentials compared to that in air, indicating that SCC occurred in all cases. To bolster the reliability of the results, each condition underwent three separate tests, confirming the repeatability of the findings. As shown in Table 4, there were no significant differences in the data. The susceptibility to SCC under OCP and various applied potentials was evaluated using the $I_{\psi }$ and $I_{\delta }$ as shown in Figure 10. The relationship between SCC susceptibility and potentiation was determined based on these parameters. It can be seen that $I_{\psi }$ and $I_{\delta }$ exhibit similar trends with respect to changes in potential [31,32].

As the electrochemical potential is shifted from the OCP to −800 mV, a significant decrease in the SCC susceptibility of X70 pipeline steel is observed. This notable reduction in susceptibility can be attributed to the efficient suppression of AD, a finding that is supported by the electrochemical impedance spectroscopy measurements showing the highest recorded R_ct._ at this potential. This indicates that the protective processes that counteract AD are more effective under these conditions, thereby reducing the likelihood of SCC occurrence in the material. However, when the electrochemical potential is adjusted further from −800 mV to −1200 mV, there is a gradual increase in SCC susceptibility. This increase is likely influenced by the phenomenon of hydrogen evolution, which tends to become more pronounced at increasingly negative potentials. As hydrogen ions are reduced to hydrogen gas, the accumulation of atomic hydrogen can lead to the embrittlement of the steel, thus enhancing SCC susceptibility [18].

At the OCP, the SCC process is predominantly governed by AD [18]. Preferential AD occurs at surface defects or slip band steps, promoting the formation of corrosion pits [33]. Under applied stress and localized acidification, these pits can serve as initiation sites for microcracks, which then propagate into the steel.

Upon the formation of SCC microcracks, the crack tip, characterized by increased electrochemical activity, serves as the anodic site. In contrast, the extensive area of the crack walls functions as the cathode. This significant galvanic effect boosts AD at the crack tip, greatly aiding in crack propagation [30]. At a potential of −800 mV, the susceptibility to SCC is considerably diminished due to the effective inhibition of AD and a lack of significant hydrogen evolution [34,35,36,37]. Conversely, when the potential decreases from −800 mV to −1200 mV, SCC susceptibility escalates rapidly. Analysis of the fast- and slow-scan polarization curves indicates that at −950 mV, mild AD occurs at the crack tip, with notable hydrogen evolution taking place at the crack walls. At potentials of −1050 mV and −1200 mV, prominent hydrogen evolution is observed at both the crack tip and the walls [18].

As the cathodic potential is decreased and becomes more negative, there is a significant increase in the local pH levels. This rise in pH leads to a heightened rate of hydrogen evolution, which is the process by which hydrogen gas is produced. At the same time, this negative shift in potential results in the formation of a dense layer of Ca and Mg deposits on the surface of the steel. These deposits create a barrier that obstructs the diffusion of oxygen, which is essential for various corrosion processes [38]. This results in a dramatic increase in mobile hydrogen content, as well as hydrogen trapped at bainitic lath boundaries. Since lath boundary separation occurs only when hydrogen concentration exceeds a certain threshold—one that depends on applied cathodic potential, exposure time, the specific size of the laths, and their hydrogen trapping capacity—more negative cathodic potentials exacerbate lath boundary decohesion [39]. This effect, in turn, increases SCC susceptibility.

### 3.4. Fracture Morphology

**Figure 11:**
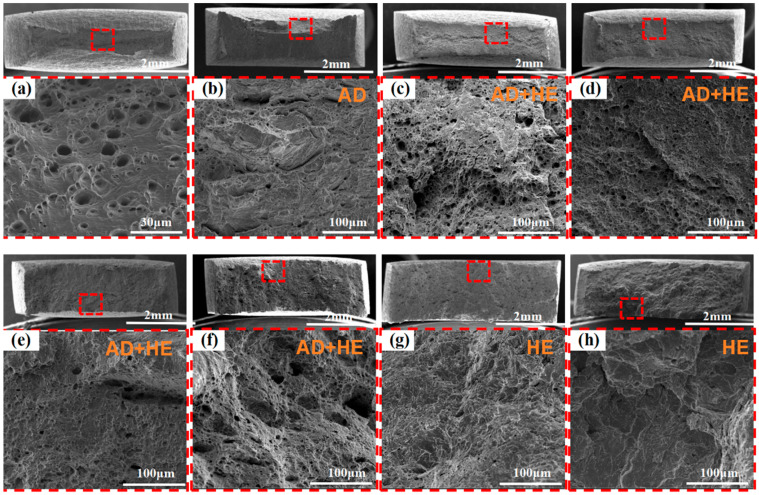
SEM morphologies of fracture surface at various potentials. (**a**) In air; (**b**) at OCP; (**c**) −800 mV; (**d**) −850 mV; (**e**) −900 mV; (**f**) −950 mV; (**g**) −1000 mV; and (**h**) −1200 mV.

**Figure 12:**
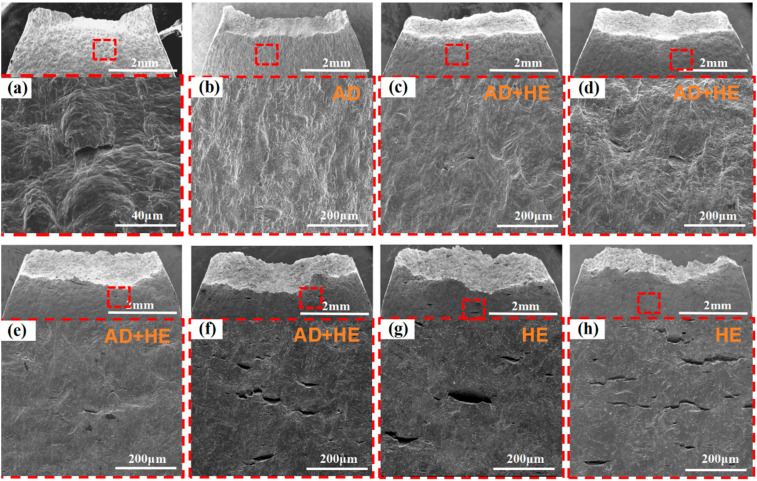
SEM morphologies of fracture side face. (**a**) In air; (**b**) at OCP; (**c**) −800 mV; (**d**) −850 mV; (**e**) −900 mV; (**f**) −950 mV; (**g**) −1000 mV; and (**h**) −1200 mV.

Figure 11 and Figure 12 present the SEM morphologies of fracture surfaces and side views obtained in air and under various applied potentials. Under OCP conditions, the fracture surfaces generally exhibit relatively ductile features, with evidence of microvoid coalescence. Although local AD may initiate pits or microcracks at surface defects, the absence of a strongly negative cathodic potential limits the severity of hydrogen evolution [25]. As a result, crack propagation is primarily governed by AD and mechanical factors, leading to lower embrittlement and more ductile-like fracture characteristics.

Previous electrochemical analyses indicate that AD is effectively suppressed at −800 mV, and hydrogen evolution is minimal. The fracture surfaces at this potential typically appear smoother, with fewer secondary cracks, reflecting an optimal protective state against both anodic dissolution and hydrogen-induced damage [40]. Within this potential range, SCC susceptibility is notably reduced.

As the potential shifts to more negative values (e.g., −850 to −950 mV), the influence of HE becomes increasingly pronounced. The fracture surfaces begin to display higher microvoid densities and subtle brittle regions. The localized accumulation of hydrogen near the crack tip promotes slight weakening, even though AD remains partially controlled. In these intermediate potential ranges, the interplay between moderated AD and gradually intensifying hydrogen uptake results in mixed-mode fracture surfaces, incorporating both ductile and brittle characteristics. Correspondingly, SCC susceptibility starts to increase.

At significantly more negative potentials (e.g., −1000 to −1200 mV), the fracture surfaces reveal quasi-cleavage facets, reduced ductility, and numerous microvoids or microcracks indicative of hydrogen-assisted crack growth. These features strongly suggest an HE mechanism. Increased hydrogen absorption and trapping within the microstructure greatly facilitate embrittlement, rendering the fracture more brittle. As the cathodic potential becomes increasingly negative, these brittle characteristics become more pronounced [39]. Under these conditions, hydrogen accumulates along bainitic lath boundaries, ultimately causing their decohesion. Under tensile stress, hydrogen rapidly migrates ahead of the crack tip, thereby accelerating crack propagation [41]. At more negative potentials, the degree of decohesion along the bainitic lath boundaries increases significantly. This is due to a larger number of hydrogen-trapping sites surpassing the critical threshold, which leads to more pronounced separation. Consequently, additional secondary cracks are detected across the fracture surface (Figure 12), significantly increasing SCC susceptibility, which is consistent with prior electrochemical and SSRT data. In summary, the fractographic morphologies observed across the potential spectrum—from OCP to strongly negative values—provide a coherent, microstructural-level explanation for the transition from AD-dominated failure at OCP to hydrogen-driven embrittlement at more negative potentials.

Under OCP and −800 mV conditions, only a few shallow secondary cracks were observed along the side. These cracks typically form at an angle of approximately 45° relative to the surface, exhibit significant propagation resistance, and are thus classified as shear-type cracks [42]. Under OCP, AD often initiates pits at surface defects or dislocation slip steps. The synergy of tensile stress and localized anodic dissolution facilitates crack initiation at these pits, eventually resulting in SCC. In contrast, at −800 mV, AD is significantly inhibited, with only minor corrosion detected, resulting in a significant reduction in SCC susceptibility. No obvious signs of corrosion were observed after testing when the applied potential exceeded −800 mV. However, the sample surface exhibited numerous wide and deep secondary cracks. Instead of extending along grain boundaries, these cracks propagate perpendicularly to the applied tensile stress. This behavior indicates a transgranular mode of SCC [43].

## 4. Discussion

### 4.1. The Stress Corrosion Cracking Mechanism of X70 Pipeline Steel

The SCC mechanism of X70 pipeline steel in simulated seawater environments is governed by the complex interactions between AD and HE, and their relative dominance is influenced by the applied potential. From the electrochemical data, the fast and slow scanning polarization curves (Figure 4) show that the dissolution current at the crack tip is significantly larger than that at the crack wall between −437 mV and −774 mV, which indicates that anodic dissolution is the dominant mechanism. And, in the range of −774 mV to −996 mV, the anodic dissolution at the crack tip and the hydrogen evolution at the crack wall occurred simultaneously, which showed a mixed control feature. When the potential is lower than −996 mV, both the crack tip and the wall undergo obvious HER, leading to the dominance of the hydrogen embrittlement mechanism. The EIS test results (Figure 5) and the Rt–potential relationship curves (Figure 8) further confirm this mechanism transition process. The maximum charge transfer resistance R_t_ value is obtained for the system at −800 mV, indicating that the best protection is obtained for the material at this time. This corresponds to the stress–strain behavior shown by the SSRT test results (Figure 9), where the material exhibits the lowest SCC susceptibility at −800 mV. At OCP and −800 mV conditions, the fracture is dominated by tough nests with only a few secondary cracks, which is consistent with the low SCC susceptibility shown by the electrochemical data. In the range of −850 mV to −950 mV, the fracture shows mixed features with both tough fracture characteristics and localized brittle regions, which is consistent with the combined effect of AD and HE in this potential range, and when the potential decreases to −1000 mV to −1200 mV, the fracture shows typical quasi-disintegration characteristics with a large number of microvoids and secondary cracks, which is consistent with the failure mode dominated by hydrogen embrittlement mechanism. The SCC susceptibility versus potential curve in Figure 10 clearly demonstrates the potential-dependent characteristics of the material properties. From this curve, it can be seen that the material exhibits the lowest SCC susceptibility at −800 mV, which corresponds to the highest R_t_ value observed in the EIS test. As the potential deviates from −800 mV in the negative direction, the SCC susceptibility gradually increases, which is consistent with the trend of the fracture morphology shift from ductile to brittle fracture. This transition can be explained by the enhanced HER observed in the electrochemical tests.

### 4.2. SCC Evaluation of X70 Pipeline Steel in Simulated Seawater Environment

Based on Liu’s report on the relationship between *I_SCC_* and non-steady electrochemical processes [12], the SCC mechanism and the associated *I_SCC_* for X70 pipeline steel in simulated seawater environments are presented as follows:

(3) $$\begin{array}{c} \begin{array}{c} I_{SCC}=\left\{\begin{array}{cccc} k_{a}\cdot i_{f}\cdot \left(\frac{i_{f}-i_{s}}{i_{s}}\right)+I_{0} & \left(i_{f}>i_{s}>0\right) & & \\ k_{he}\cdot \left|i_{s}\right|+k_{ad}\cdot i_{f}\cdot \left|\frac{i_{f}}{i_{s,corr}}-1\right|+I_{ac} & \left(i_{f}>0,i_{s}<0\right) & & \\ k_{c}\cdot \left|i_{s}\right|+I_{c} & \left(i_{f}<0,i_{s}<0\right) & & \end{array}\right. \end{array} \end{array}$$

where *k_a_*, *k_he_*, *k_ad,_* and *k_c_* denote constants that vary with the current density, corrosion medium, and material. The current densities obtained from the slow-sweep and fast-sweep polarization curves are expressed as *i_s_* and *i_f_*, respectively, where the corrosion current density measured from the slow-sweep curve is denoted as *i_s,corr_*. Once the condition *i_s_* = *i_s,corr_* is satisfied, the standard stress corrosion cracking index, *I_0_*, which characterizes the anodic dissolution process, can be determined. Moreover, the parameter *I_ac_* comprehensively captures the synergistic effects of anodic dissolution and hydrogen embrittlement, whereas *I_c_* represents the standard *I_SCC_* predominantly driven by hydrogen embrittlement under identical experimental conditions [44].

**Figure 13:**
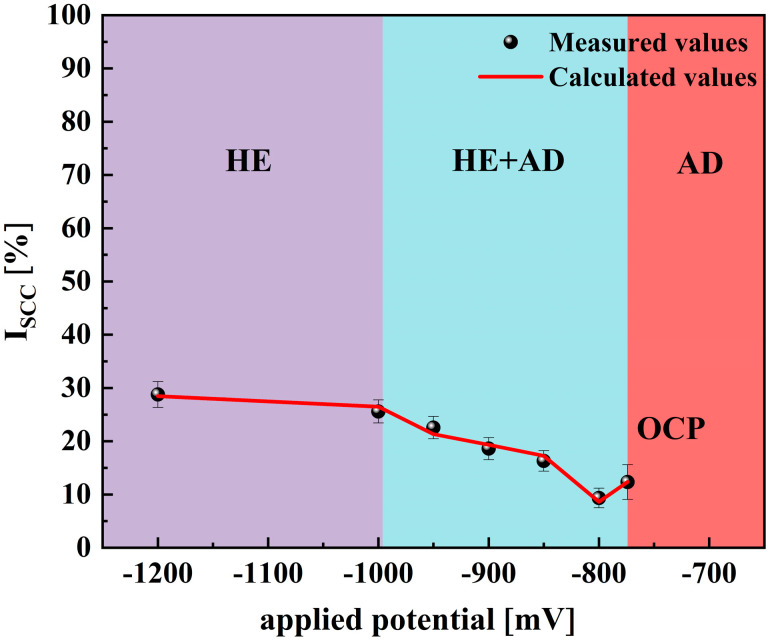
The measured SCC susceptibility values and the calculated values of X70 in simulated seawater environments.

While the original model was developed specifically for X70 pipeline steel in acidic soil, this study focuses on characterizing its SCC behavior in a marine environment. We found that the model is also applicable to X70 pipeline steel in marine environments. We calculated the coefficients and constants by linear interpolation based on Equation (3) and the measured stress corrosion susceptibility values to derive the relationship between stress corrosion susceptibility and applied potential. It should be noted that we used the area shrinkage loss ($I_{\psi }$) values, as $I_{\psi }$ is more suitable for characterizing the SCC propagation trend. Figure 13 shows the measured and calculated values of stress corrosion susceptibility, which are in good agreement. Thus, the non-stationary electrochemical model is equally accurate in determining the SCC mechanism and predicting the SCC susceptibility of X70 steel in marine environments with applied polarization potentials.

## 5. Conclusions

(1) In this study, it was found that the unsteady-state electrochemical model is also applicable to the rapid assessment of the stress corrosion of X70 pipeline steel under marine conditions. Relying only on electrochemical tests and a limited number of SSRT experiments to rapidly assess SCC susceptibility over the entire potential range, the model is easy to use and highly accurate.
(2) X70—the mechanism of SCC non-stationary electrochemistry in simulated seawater environments depends on the applied potentials. Between −437 mV and −774 mV, SCC susceptibility is low and is controlled by AD. Between −774 mV and −996 mV, the increase in SCC susceptibility shifts to be controlled by both AD and HE, and when the potential falls below −996 mV, SCC susceptibility increases dramatically and is controlled by HE.
(3) The model offers great potential for application in pipeline design, maintenance planning, and risk assessment frameworks, thus contributing to safer and more economically viable offshore infrastructure. Future research should focus on extending this approach to a wider range of pipeline steels such as X52, X65, and X100 to determine its general applicability.

## Figures and Tables

**Table 1:** The chemical composition of X70 pipeline steel (wt%).

C	Si	Mn	P	S	Cr	Ni	Mo	Nb	V	Ti	Al	Fe
0.044	0.26	1.13	0.004	0.002	0.27	0.13	0.16	0.06	0.02	0.015	0.005	Bal.

**Table 2:** Mechanical Properties of X70 Pipeline Steel.

Elongation [%]	Yield Strength [MPa]	Tensile Strength [MPa]	Impact Energy [J]	Brinell Hardness [HB]
≥16	≥485	570–760	≥120	≤255

**Table 3:** Chemical composition of simulated seawater (g/L).

NaCl	MgCl_2_	Na_2_SO_4_	CaCl_2_	KCl	NaHCO_3_	KBr	H_2_BO_3_
24.53	5.2	4.09	1.16	0.695	0.201	0.101	0.027

**Table 4:** SSRT results under each condition.

Conditions	Specimen Number	Area Reduction [%]	$I_{\psi }$ [%]	Elongation [%]	$I_{\delta }\;$ [%]	Yield Strength [MPa]	Tensile Strength [MPa]
In air	1#	53.61		17.64		508.96	595.75
	2#	54.24		17.5		505.61	591.03
	3#	52.89		17.93		506.34	594.17
At OCP	4#	43.9	19.99 ± 2.36	13.38	23.19 ± 1.21	473.27	563.84
	5#	43.3		13.47		476.95	567.52
	6#	41.47		13.79		469.76	558.91
−800 mV	7#	51.03	5.47 ± 0.94	16.13	9.34 ± 1.84	486.07	546.57
	8#	50.68		15.61		487.64	550.79
	9#	50.31		16.23		489.40	552.88
−850 mV	10#	49.12	9.51 ± 1.17	15.03	16.30 ± 1.93	492.94	560.58
	11#	48.53		14.38		493.87	562.35
	12#	47.88		14.88		490.76	558.29
−900 mV	13#	46.94	14.16 ± 1.73	14.45	18.60 ± 2.08	495.07	573.60
	14#	46.04		13.95		493.79	572.15
	15#	45.07		14.67		492.33	570.94
−950 mV	16#	44.17	19.70 ± 1.96	13.63	22.56 ± 2.10	492.45	569.23
	17#	42.90		13.30		491.18	567.08
	18#	42.08		14.04		493.93	569.83
−1000 mV	19#	37.26	33.87 ± 3.61	13.17	25.59 ± 2.16	488.47	563.63
	20#	35.68		12.72		486.82	561.70
	21#	33.40		13.48		488.29	562.95
−1200 mV	22#	27.49	52.32 ± 3.8	12.62	28.77 ± 2.43	479.36	560.14
	23#	25.83		12.11		482.05	562.96
	24#	23.36		12.97		481.87	561.77

## Data Availability

The original contributions presented in this study are included in the article. Further inquiries can be directed to the corresponding authors.

## Abbreviations

SCC	Stress corrosion cracking
AD	Anodic dissolution
HE	Hydrogen embrittlement
CP	Cathodic protection
OCP	Open circuit potential
DFT	Density functional theory
EIS	Electrochemical impedance spectroscopy
SSRT	Slow strain rate testing
SEM	Scanning electron microscope
SE	Secondary electron
WD	Working distance
HER	Hydrogen evolution reaction
R_s_	Solution resistance
R_f_	Film resistance
Q_f_	Film constant phase element
Q_dl_	Double-layer constant phase element
R_t_	Charge transfer resistance
R_ta_	Anodic charge transfer resistance
R_tc_	Cathodic charge transfer resistance
R_tO_	Charge transfer resistance of oxygen reduction reaction
R_tH_	Charge transfer resistance of hydrogen evolution reaction
$I_{\delta }$	SCC susceptibility (the loss rate of elongation)
$I_{\psi }$	SCC susceptibility (the reduction rate of area)
$\delta_{0}$	The elongation measured in air
$\delta_{s}$	The elongation measured in various potentials
$\psi_{0}$	The reduction in the area measured in air
$\psi_{s}$	The reduction in the area measured in various potentials
*K_a_*_,_ *K_he_*_,_ *K_ad_*_,_ *K_c_*	A constant that varies with the current density, corrosion medium, and material
*i_s_*	Current density obtained from the slow-sweep polarization curve
*I_f_*	Current density obtained from the fast-sweep polarization curve
*i_s,corr_*	Corrosion current density measured from the slow-sweep curve
*I* *_0_*	The standard SCC index
*I_ac_*	A parameter that captures the synergistic effects of anodic dissolution and hydrogen embrittlement
*I_c_*	The standard SCC index dominated by hydrogen embrittlement under identical experimental conditions
*I_SCC_*	The standard SCC index
